# A Mouse Model of Creatine Transporter Deficiency Reveals Impaired Motor Function and Muscle Energy Metabolism

**DOI:** 10.3389/fphys.2018.00773

**Published:** 2018-06-22

**Authors:** Malte Stockebrand, Ali Sasani, Devashish Das, Sönke Hornig, Irm Hermans-Borgmeyer, Hannah A. Lake, Dirk Isbrandt, Craig A. Lygate, Arend Heerschap, Axel Neu, Chi-Un Choe

**Affiliations:** ^1^German Center for Neurodegenerative Diseases, Bonn, Germany; ^2^Institute for Molecular and Behavioral Neuroscience, University of Cologne, Cologne, Germany; ^3^Department of Neurology, University Medical Center Hamburg-Eppendorf, Hamburg, Germany; ^4^Experimental Neuropediatrics, Department of Pediatrics, University Medical Center Hamburg-Eppendorf, Hamburg, Germany; ^5^Department of Radiology, Radboud University Nijmegen Medical Center, Nijmegen, Netherlands; ^6^Transgenic Mouse Unit, Center for Molecular Neurobiology Hamburg, University Medical Center Hamburg-Eppendorf, Hamburg, Germany; ^7^Division of Cardiovascular Medicine, Radcliffe Department of Medicine, BHF Centre of Research Excellence, University of Oxford, Oxford, United Kingdom; ^8^Wellcome Centre for Human Genetics, University of Oxford, Oxford, United Kingdom

**Keywords:** creatine transporter, skeletal muscle, energy metabolism, *in vivo* magnetic spectroscopy, L-arginine:glycine amidinotransferase (AGAT), guanidinoacetate *N*-methyltransferase (GAMT), glycine amidinotransferase (GATM), Slc6a8

## Abstract

Creatine serves as fast energy buffer in organs of high-energy demand such as brain and skeletal muscle. L-Arginine:glycine amidinotransferase (AGAT) and guanidinoacetate *N*-methyltransferase are responsible for endogenous creatine synthesis. Subsequent uptake into target organs like skeletal muscle, heart and brain is mediated by the creatine transporter (CT1, SLC6A8). Creatine deficiency syndromes are caused by defects of endogenous creatine synthesis or transport and are mainly characterized by intellectual disability, behavioral abnormalities, poorly developed muscle mass, and in some cases also muscle weakness. CT1-deficiency is estimated to be among the most common causes of X-linked intellectual disability and therefore the brain phenotype was the main focus of recent research. Unfortunately, very limited data concerning muscle creatine levels and functions are available from patients with CT1 deficiency. Furthermore, different CT1-deficient mouse models yielded conflicting results and detailed analyses of their muscular phenotype are lacking. Here, we report the generation of a novel CT1-deficient mouse model and characterized the effects of creatine depletion in skeletal muscle. HPLC-analysis showed strongly reduced total creatine levels in skeletal muscle and heart. MR-spectroscopy revealed an almost complete absence of phosphocreatine in skeletal muscle. Increased AGAT expression in skeletal muscle was not sufficient to compensate for insufficient creatine transport. CT1-deficient mice displayed profound impairment of skeletal muscle function and morphology (i.e., reduced strength, reduced endurance, and muscle atrophy). Furthermore, severely altered energy homeostasis was evident on magnetic resonance spectroscopy. Strongly reduced phosphocreatine resulted in decreased ATP/Pi levels despite an increased inorganic phosphate to ATP flux. Concerning glucose metabolism, we show increased glucose transporter type 4 expression in muscle and improved glucose clearance in CT1-deficient mice. These metabolic changes were associated with activation of AMP-activated protein kinase – a central regulator of energy homeostasis. In summary, creatine transporter deficiency resulted in a severe muscle weakness and atrophy despite different compensatory mechanisms.

## Introduction

Energy supply for muscle contraction is provided by breakdown of adenosine triphosphate (ATP), which is replenished continuously by anaerobic glycolysis or oxidative phosphorylation. The (P)Cr system serves as rapid energy buffer to maintain constant ATP levels and mediates subcellular high-energy phosphate transfer. Therefore, high (P)Cr levels are found in skeletal muscle in concentrations up to 20–40 mM (Wyss and Kaddurah-Daouk, 2000). As non-enzymatic degradation of creatine to creatinine takes place at a daily rate of 1.7% (Wyss and Kaddurah-Daouk, 2000), the Cr pool has to be replenished via dietary intake or endogenous Cr synthesis. AGAT (EC 2.1.4.1) and GAMT (EC 2.1.1.2) are responsible for Cr synthesis from arginine, glycine, and *S*-adenosyl-methionine, which mainly takes place in kidney and liver. Subsequent uptake of Cr into organs such as brain, heart, and muscle is mediated by the Cr transporter (CT1, SLC6A8).

Defects of Cr synthesis or transport due to mutations in *AGAT*, *GAMT*, or *SLC6A8* (*CT1*) genes are associated with Cr deficiency syndromes that affect brain and muscle to various degrees (Stockler et al., 2007). Patients with CT1 deficiency are especially prone to intellectual disability, behavioral abnormalities and seizures. Mutations in *SLC6A8* are relatively frequent and were found in up to 2% of cases of X-linked intellectual disability (van de Kamp et al., 2014). Slender habitus and poorly developed muscle mass are the most striking anthropometric features of CT1 deficiency. Motor dysfunction can affect CT1-deficient patients, with muscular hypotonia in 10% and, less common, unspecific myopathic symptoms (Mancini et al., 2005; Puusepp et al., 2010; van de Kamp et al., 2014, 2015). Significant amounts of Cr were still detectable in the skeletal muscle of examined patients and studied knockout mice. However, a significant reduction of muscle Cr was only visible in mouse models. The relatively preserved Cr levels in skeletal muscle of CT1-deficient patients may possibly be due to endogenous Cr synthesis, non-specific uptake or residual activity of patients’ mutant CT1 (deGrauw et al., 2003; Pyne-Geithman et al., 2004; Russell et al., 2014; Baroncelli et al., 2016).

We previously generated and characterized mouse models with AGAT and GAMT deficiency (Schmidt et al., 2004; Choe et al., 2013b). In addition to pronounced CNS and metabolic phenotypes, these mice displayed impaired muscle strength and muscle atrophy underlining the role of the (P)Cr system in skeletal muscle (Torremans et al., 2005; Nabuurs et al., 2013). Previous studies on CT1-deficient mouse models have focused on the central nervous system. The muscle phenotype has not yet been analyzed in detail and the preliminary characterization generated conflicting results from different models and methodological approaches (Skelton et al., 2011; Russell et al., 2014; Baroncelli et al., 2016). Therefore, we generated and characterized CT1-deficient mice with a focus on muscle physiology. Our novel CT1-deficient mouse model revealed (P)Cr deficiency in skeletal muscle associated with impaired motor function and muscle atrophy, which is in agreement with previous models (Russell et al., 2014; Baroncelli et al., 2016). Magnetic resonance spectroscopy experiments were performed to elucidate the high-energy phosphate metabolism in skeletal muscle of CT1-deficient mice. Furthermore, glucose metabolism, enzymes of creatine biosynthesis, and proteins of oxidative phosphorylation were studied in skeletal muscle of CT1-deficient mice to elucidate altered pathways.

## Materials and Methods

### Ethics Statement

All animal procedures were conducted in accordance with institutional and national guidelines and were approved by the respective local animal ethics committees (Hamburg: 11/89 and 12/86, Nijmegen: RU-DEC2012-184). All experimental procedures conformed with the Guide for the Care and Use of Laboratory Animals published by the US National Institutes of Health (NIH Publication No. 83-123, revised 1996) and were performed in accordance with the ARRIVE guidelines. Mice used in this study were obtained from heterozygous matings. CT1-deficient knockout (CT1^-/y^) mice and controls were age- and sex-matched littermates. Mice (<5 per cage) were kept in standard cages under a 12–12 h light–dark cycle, constant temperature, and humidity, and received standard food and water *ad libitum*. Chow was essentially Cr free (R/M-H, Ssniff), as previously shown (Schmidt et al., 2004).

### Generation of CT1-Deficient Mice

In brief, CT1-deficient knockout (CT1^-/y^) mice were generated by injection of embryonic stem cells (ESCs) containing a targeted *CT1* (*Slc6a8*) locus obtained from the NIH Knock-out Mouse Program (KOMP, project CSD24513, *Slc6a8*^tm1a(KOMP)Wtsi^ allele in ES cell clone EPD0293_6_E05). The ESCs from the parental line JM8A1.N3 (C57Bl/6N derived; Pettitt et al., 2009) had been targeted with a targeting-vector (PRPGS00081_A_A09) consisting of all 13 *Slc6a* exons with loxP sites flanking exons 5–8 (Skarnes et al., 2011). The targeted allele contains an insertion of a beta-galactosidase/lacZ reporter expression cassette (L1L2_Bact_P) with a splice acceptor site in the intron between exons 4 and 5, for the detection of Slc6a8 gene expression. The ESC clone was expanded and microinjected into C57BL/6J mouse blastocysts, which were then transferred into pseudo-pregnant C57BL/6J females. Chimeric mice were mated and gave rise to germ-line transmission of the disrupted allele. Mice were backcrossed for several generations to C57BL/6J genetic background with negative selection for the Rd8 mutation in the F1 generation. Genomic DNA from mouse-tail or ear biopsies was screened by multiplex polymerase chain reaction (PCR) following standard protocols. Sequencing of genomic PCR-products between exons 7 and 8 revealed absence of the 3′ loxP site from the conditional *Slc6a8*^tm1a(KOMP)Wtsi^ allele. Therefore, according to the KOMP mutagenesis scheme, the correct description of the targeted, non-conditional allele is *Slc6a8*^tm1e(KOMP)Wtsi^ knock out first allele.

### cDNA Preparation and PCR

**FIGURE 1:**
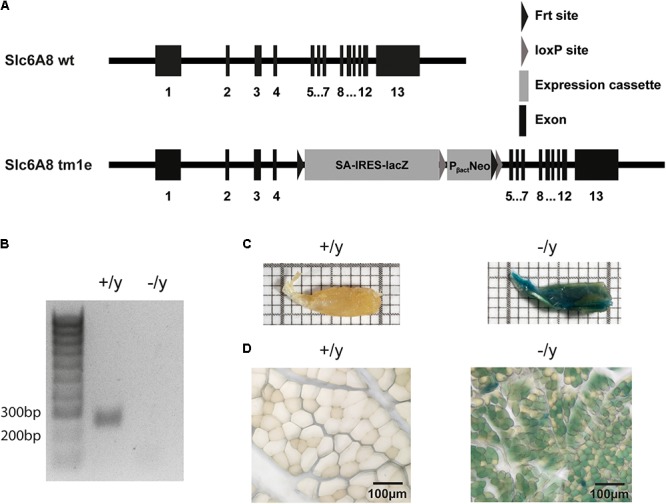
Generation and characterization of CT1-deficient mice. **(A)** The CT1 gene was disrupted by insertion of a SA-IRES-lacZ-neomycin cassette (tm1e allele). **(B)** Total RNA prepared from muscle of CT1^+/y^ and CT1^-/y^ mice was reverse transcribed and then used for PCR amplification with primers specific for CT1 (expected band size: 250 bp). No amplification product for CT1 was observed in knockout mice. **(C,D)** Macroscopic (on a mm grid scale) and microscopic view of hind limb muscle after LacZ staining indicating CT1 promoter activation.

Disruption of the murine *CT1* gene was confirmed by touch down PCR of skeletal muscle cDNA using primers binding in exons 7 and 9 (**Figure 1B**). Total RNA of knock-down and wild-type mice was isolated using TRIzol reagent (Invitrogen) according to the manufacturer’s instructions. DNase I treatment was performed to remove genomic DNA. One microgram of total RNA was used for random primed reverse transcription using the First Strand cDNA Synthesis Kit (Thermo Scientific) according to standard protocols. cDNAs were stored at -20°C. Exon spanning primers (forward: 5′-tcctggcactcatcaacag-3′, reverse: 5′-atgaagccctccacacctac-3′) were taken from Ireland et al. (2009) expecting a 250 bp PCR product in wild-type reaction. Touch down PCR was performed using the following protocol: annealing temperature was decreased in 12 steps from 60°C by 0.5°C to 54°C followed by 25 cycles at 54°C.

### Rotarod Test

Mice had to walk on a turning, corrugated rod (3.2 cm in diameter) (Acceler. Rotarod for mice, Jones & Roberts, TSE 308 systems, Bad Homburg, Germany). The rod was started to rotate 5 s after the mice were placed onto it. Mice underwent five trials with an inter-trial-interval (ITI) of 45 min, and performed at slow, constant speed [i.e., 4 rotations per minute (rpm)] for a maximum duration of 3 min. Trials 3–5 were performed with the accelerating rod, starting with 4 rpm up to 40 rpm within 4 min, with a maximum duration of 10 min. On the following day, a sixth trial was carried out with the accelerating rod. The performance of the mice was evaluated by scoring the latency to fall. Experiments were performed blinded.

### Pole Test

The animals were placed on top of a vertical 48.5 cm-long rod made of rough wood with a diameter of 0.8 cm. To motivate the mouse to climb down, nesting material of the home cage was placed at the bottom of the pole. The mouse was placed with its head facing upward and its four paws grasping the rod. The time required by the mouse to reach the floor (maximum duration of each trial was 80 s) was recorded. Each mouse had to perform three consecutive trials with an ITI of 30 s. The ability of a mouse to turn 180° and climb down with the head pointing downward was evaluated. When the mouse turned, we recorded whether it turned at the top (level 1, above 32 cm), at the middle (level 2, between 32 and 16 cm), or at the bottom of the rod (level 3, below 16 cm). Experiments were performed blinded.

### Grip Strength

Maximal grip force was measured using a grip strength meter (TSE-Systems, Bad Homburg, Germany) as described previously (Nabuurs et al., 2013). Within each group, the mean grip force of each mouse was calculated from 15 appropriate trials. Experiments were performed blinded.

### Voluntary Running Wheel

Mice were provided with a vertical, ridged running wheel (tierisch.de, Flensburg, Germany). The wheels were fixed in the cage using magnets. Wheel rotation was monitored using a magnetic read switch attached to a counter. Running distances were calculated based on the number of rotations within 24 h and the circumference of the running wheel (41.41 cm). Experiments were performed blinded.

### Muscle Histopathology

Muscle histology was performed as previously described (Nabuurs et al., 2013). Briefly, mice were transcardially perfused with 4% formaldehyde in PBS. For morphometry, commonly stained slides for hematoxylin and eosin (H&E) were used. Myocyte diameters and CSA were determined in multiple transverse sections of muscle using ImageJ and calculated by averaging the data of five images. Determination of myocyte diameters was performed blinded.

### *In Vivo* ^1^H MRI and ^31^P MRS

Male CT1^+/y^ and CT1^-/y^ mice, aged 120–180 days, were placed in prone position in the magnet bore under anesthesia with 1–2% isoflurane in a 2:1 air/O_2_ mixture and maintained at 37 ± 1°C using warm air under control of a rectal sensor (Luxtron 712, Santa Clara, CA, United States). Breathing rate (50–70/min) was monitored by a respiratory pad attached to the chest. Measurements were performed on an 11.7T Biospec Avance III small animal MR system (Bruker, Biospin, Germany). The hind legs of mice were placed in a home-built 8 mm diameter radiofrequency (RF) probe consisting of a ^1^H and a ^31^P coil. Gradient-echo localizer MR images were obtained in three orthogonal directions followed by localized magnetic field shimming. Then a 2D gradient-echo image was obtained of a 2 mm thick transversal slice, positioned at the largest CSA distal from the knee joint (FOV = 2 cm × 2 cm, matrix size = 128 × 128, resolution = 0.156 mm × 0.156 mm, TR = 100 ms, TE = 6 ms). Non-localized ^31^P MR spectra of the leg (gastrocnemius/plantaris/soleus complex) were recorded using an excitation block pulse of 100 μs with a 45° flip angle, a 8012 Hz sweep width, 4096 data points, 256 ms acquisition time, TR 5 s, 128 averages for CT1^+/y^ and 400–512 averages for CT1^-/y^ mice. ^31^P saturation transfer (ST) MRS experiments were performed to derive the Pi→ATP (pseudo first order) forward rate constant *k*_f_ in the leg muscles (Nabuurs et al., 2010, 2013) using Gaussian frequency selective saturation pulses (1000 points, duration 100 ms, selectivity ∼50 Hz) with pulse power optimization for complete resonance saturation (∼370 rad/sec). Saturation was applied at the γATP frequency and its mirror frequency around the Pi peak using looped incremental saturation periods of 0.1, 0.2, 0.4, 0.6, 1.0, 1.5, and 3 s (Koretsky et al., 1985; Nabuurs et al., 2010). Total TR was 5 s (2 s TR + 3 s ST block) with 128 (CT1^+/y^) or 512 (CT1^-/y^) averages.

From the single slice 2D-gradient echo MR images the muscle CSA of the lower hind leg (mostly gastrocnemius-plantaris-soleus) of CT1^+/y^ (*n* = 5) and CT1^-/y^ (*n* = 4) was determined including a correction of the area occupied by bone (2 mm^2^). From this corrected CSA and the βATP peak integral the ATP tissue concentration in CT1^-/y^ mice was estimated assuming similar load of the RF coil by the hind legs of both mice and a tissue [ATP] of 7.8 mM for CT1^+/y^ mice (in ’t Zandt et al., 2003; Nabuurs et al., 2013). The assessment of the integrals of peaks and tissue pH from the PCr-Pi chemical shift in the ^31^P MR spectra were done as described before (Nabuurs et al., 2010, 2013). From the PCr/ATP and P_i_/ATP signal ratios we estimated tissue levels of PCr and P_i_ after correction for T1 saturation. For P_i_ a T1 of 1.95 s was applied (Nabuurs et al., 2010; Tyler et al., 2010). T1 values, for PCr and βATP, derived from skeletal muscle of 3 CT1^+/y^ mice at 11.7T using inversion recovery, were 2.25 ± 0.118 and 0.656 ± 0.07 s, respectively. The muscle Pi → ATP flux was obtained as the *k*_f_ x [P_i_] product (Nabuurs et al., 2013).

### Determination of Total Creatine

Total Cr levels were measured by HPLC from homogenized samples with normalization to non-collagen protein, adapted from Teerlink et al. (1993), as described previously (Zervou et al., 2016).

### Glucose Tolerance Tests

Mice were fasted for 14–16 h and intraperitoneally injected with glucose (2 g per kg body weight). Blood glucose was measured from tail capillary blood and collected at indicated times, as previously described (Choe et al., 2013b). Experiments were performed blinded.

### Tissue Collection and Preparation

Animals were anesthetized with an intraperitoneal injection of ketamine and xylazine or by 2–3% isoflurane in 100% oxygen. Muscles were removed and flash frozen in liquid nitrogen. Lysates were prepared in homogenization buffer containing phosphatase inhibitors with disposable 1.5-mL pestles (VWR) and cleared by centrifugation (Choe et al., 2013b). Protein concentrations were determined using a BCA^TM^ protein assay (Thermo Scientific).

### Western Blots

Equal amounts of cleared lysates (20–40 μg protein) were separated by SDS-PAGE on precast gels (Invitrogen) and transferred to nitrocellulose membrane. Antibodies against AGAT (Santa Cruz), GAMT (Eurogentec), GLUT4 (Acris Antibodies), Actin (Sigma Aldrich), UCP3 (Abcam), COX4 (Life technologies), pAMPK, pACC, and AMPK (all Cell Signaling) were used according to the manufacturers’ protocols. Enhanced chemiluminescence (Luminata Crescendo, Millipore) signals were detected with a luminescent image analyzer (LAS-4000, FujiFilm). Total protein was measured with a laser scanner (FLA-9000 with LPG filter, FujiFilm) on blot membranes stained with Lava Purple (Fluorotechnics, Gel Company) according to the manufacturer’s instructions. Signal quantification was performed on non-saturated images using ImageJ software.

### Statistical Analysis

Data are given as mean ± SEM. The following statistical tests were applied: parametric *t*-test, one-way ANOVA or two-way ANOVA followed by Newman–Keuls *post hoc* comparisons (GraphPad Prism). All tests were two-tailed and the level of significance was set at *P* < 0.05.

## Results

### Reduced Creatine Levels in Skeletal Muscle of CT1-Deficient Mice

Targeted disruption of the *Slc6a8* gene in ESCs was employed to prevent functional expression of CT1 protein (**Figure 1A**). *CT1*^-/y^ mice were born at the expected Mendelian ratio, were viable and did not require any specific treatment for survival or growth. In cDNA prepared from total RNA of skeletal muscle no expression of the *CT1* gene could be detected in *CT1*^-/y^ mice with gene specific PCR whereas a PCR product was observed in *CT1*^+/y^ mice (**Figure 1B**). HPLC-analysis revealed a 10-fold reduction of Cr levels in heart and skeletal muscle, i.e., *extensor digitorum longus* and *soleus* muscle (**Table 1**). Cerebral Cr levels were also reduced, but only by about 70% (**Table 1**). No difference of Cr levels was observed in kidney, which is the main organ of endogenous Cr biosynthesis (**Table 1**). Expression of LacZ revealed widespread activity of *CT1* promoter in skeletal muscle (**Figures 1C,D**). Thus, we successfully generated a CT1-deficient mouse line for muscle phenotype analysis.

**Table 1:** HPLC-analysis of total creatine concentrations (mean ± SEM) in CT1^+/y^ and CT1^-/y^ mice (*n* = 4 per tissue) (*t*-test, ^∗∗∗^*P* < 0.001).

Tissue	Total creatine (nmol/mg protein)	
	CT1^+/y^	CT1^-/y^
Heart	64.3 ± 6.6	4.5 ± 0.6^∗∗∗^
Brain	72.6 ± 9.5	22.5 ± 1.1^∗∗∗^
Skeletal muscle (*extensor digitorum longus*)	131.9 ± 6.7	16.1 ± 5.3^∗∗∗^
Skeletal muscle (*soleus*)	137.3 ± 9.9	15.7 ± 3.8^∗∗∗^
Kidney	12.3 ± 1.9	11.9 ± 3.8


### Cr Biosynthesis in Skeletal Muscle of CT1-Deficient Mice

**FIGURE 2:**
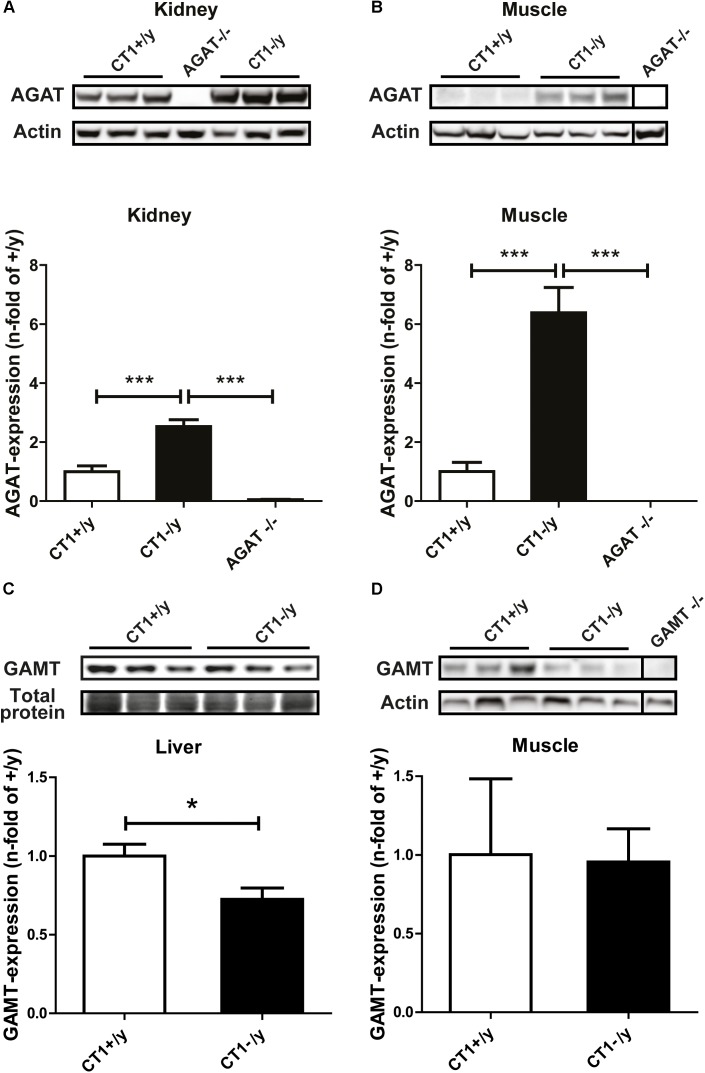
L-Arginine:glycine amidinotransferase and GAMT expression. Relative quantification of AGAT protein levels in kidney **(A)** and muscle **(B)** of CT1^-/y^ mice (*n* = 5–6). Relative quantification of GAMT protein levels in liver **(C)** and muscle **(D)** of CT1^-/y^ mice (*n* = 6). Tissue samples of AGAT^-/-^ and GAMT^-/-^ mice to verify antibody specificity (^∗^*P <* 0.05; ^∗∗∗^*P <* 0.001).

In rodents, only very low AGAT and GAMT activities were found in skeletal muscle (Van Pilsum and Warhol, 1963; Daly, 1985). However, Cr deficiency has been shown to alter AGAT protein expression. For example, GAMT-deficient mice display compensatory upregulation of AGAT expression in kidney (Choe et al., 2013a). Furthermore, AGAT expression in skeletal muscle was increased in another mouse model of CT1 deficiency (Russell et al., 2014). Therefore, we studied AGAT and GAMT protein expression in CT1-deficient mice for putative adaptive changes. Quantitative western blot analysis revealed significant upregulation of AGAT expression in kidney and skeletal muscle of *CT1*^-/y^ mice compared to wild-type mice (**Figures 2A,B**). Furthermore, well-detectable GAMT expression was unaltered in skeletal muscle of *CT1^-^*^/y^ mice, whereas GAMT expression in liver was even reduced in *CT1^-^*^/y^ mice compared with *CT1*^+/y^ mice (**Figures 2C,D**). However, despite the strong upregulation of AGAT protein expression, our data revealed pronounced reduction of Cr in skeletal muscle of *CT1^-^*^/y^ mice. Thus, increased AGAT expression in skeletal muscle does not compensate for the deficiency in skeletal muscle in *CT1^-^*^/y^ mice.

### CT1-Deficient Mice Reveal Reduced Weight, Length, and Body Mass Index

**FIGURE 3:**
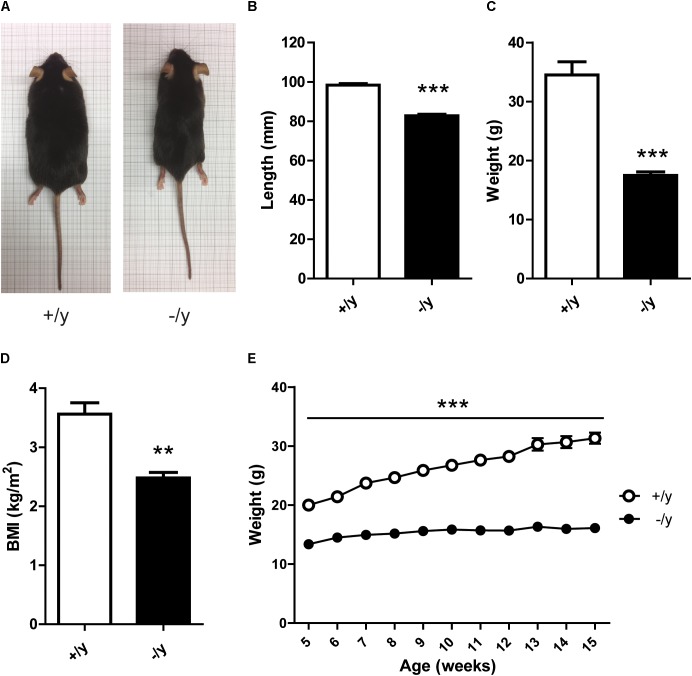
Morphometric parameters of CT1-deficient mice. Representative body morphology **(A)**, snout-anus length **(B)**, weight **(C)**, and BMI **(D)** of 15-week-old CT1^+/y^ and CT1^-/y^ male littermates (*n =* 5–6). **(E)** Body weight development in male animals until 15 weeks of age (*n* = 9–15) (^∗∗^*P <* 0.01; ^∗∗∗^*P <* 0.001).

L-Arginine:glycine amidinotransferase and GAMT deficiency in transgenic mice leads to Cr-dependent failure to thrive with reduced body weight and length (Schmidt et al., 2004; Choe et al., 2013b). Similarly, *CT1*^-/y^ mice were lean, short, and had a markedly reduced body mass index (BMI) compared with *CT1*^+/y^ littermates (**Figures 3A–D**). *CT1*^-/y^ mice showed consistently lower body weight as early as from the age of 5 weeks (**Figure 3E**). Overall, *CT1*^-/y^ mice displayed morphometric alterations indicative of severely impaired whole-body energy homeostasis.

### Impaired Motor Behavior in CT1-Deficient Mice

**FIGURE 4:**
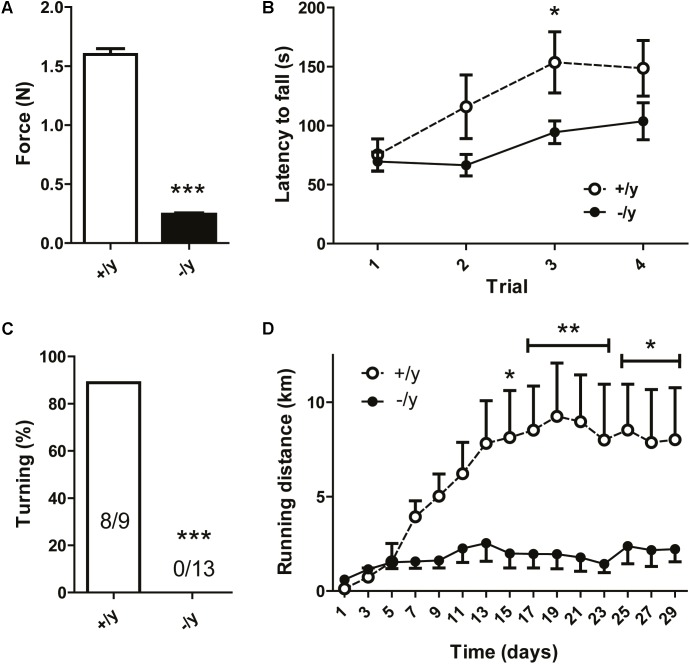
Motor and behavioral phenotype of CT1-deficient mice. Grip strength **(A)**, rotarod performance **(B)**, successful turning in pole test **(C)**, and voluntary running distance **(D)** of CT1^+/y^ and CT1^-/y^ male littermates (*n* = 9–15) (^∗^*P* < 0.05; ^∗∗^*P* < 0.01; ^∗∗∗^*P* < 0.001).

Creatine transporter-deficient mice displayed reduced body tension and severe kyphoscoliosis, suggesting pronounced muscle weakness and chronic muscular hypotonia. Therefore, we analyzed muscle function and motor behavior in detail. Grip strength of *CT1*^-/y^ mice was severely reduced to about 15% of that of *CT1*^+/y^ littermates (grip strength: *CT1*^+/y^ 1.63 ± 0.05 mN, CT1^-/y^ 0.25 ± 0.01 mN, *n* = 9–15, **Figure 4A**). In the rotarod test, latency to fall did not differ between the two groups in the first trial. However, *CT1*^+/y^ littermates showed motor learning and increased performance during subsequent trials, whereas *CT1*^-/y^ mice remained at a constantly low level (**Figure 4B**). During the pole test, *CT1*^-/y^ mice were not capable of turning upside down, whereas normal performance was observed in almost all *CT1*^+/y^ littermates (turning: *CT1*^+/y^: 8/9 mice, *CT1*^-/y^: 0/13 mice, **Figure 4C**). In addition to strength, motor learning, and coordination, we analyzed running endurance using an adapted voluntary wheel-running system (De Bono et al., 2006). In contrast to *CT1*^+/y^ mice, the running distance of *CT1*^-/y^ mice was dramatically reduced (running distance: *CT1*^+/y^ 8.41 ± 0.85 km/day, *CT1*^-/y^ 1.99 ± 0.26 km/day, average of days 15–29, **Figure 4D**). Taken together, *CT1*^-/y^ mice displayed a motor phenotype characterized by severe muscular hypotonia and kyphoscoliosis, reduced muscle strength and low running endurance.

### Muscle Atrophy in CT1-Deficient Mice

**FIGURE 5:**
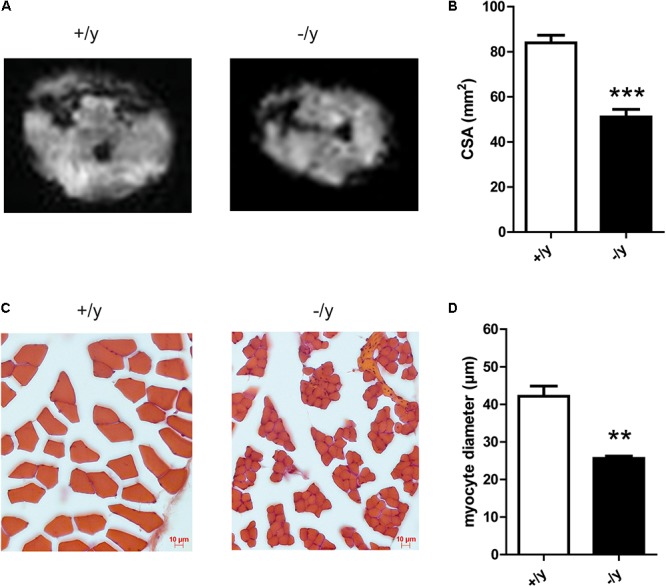
Muscle morphology and histology of CT1-deficient mice. **(A)** MR images for gross morphological analysis of hind limb muscles. **(B)** Cross-sectional area of hind limb muscle (*n* = 4–5, ^∗∗∗^*P* < 0.001) determined by MRI. **(C)** Representative histological staining of extensor digitorum longus muscle. **(D)** Myocyte diameter of extensor digitorum longus muscle (*n* = 3, ^∗∗^*P <* 0.01).

We analyzed the underlying cause of impaired motor function of *CT1*^-/y^ mice at MRI and histological levels. MRI of the hind leg revealed reduced muscle CSAs in *CT1*^-/y^ mice (*CT1*^+/y^ 83.9 ± 3.0 mm^2^, *CT1*^-/y^ 51.0 ± 3.0 mm^2^, *n* = 4–5, **Figures 5A,B**). We then performed muscle histology to look if potential cell loss or atrophy explains reduced muscle size. Single fiber atrophy was detected in the *extensor digitorum longus* muscle, where quantitative analysis revealed reduced myocyte CSAs in *CT1*^-/y^ mice compared with *CT1*^+/y^ mice (*CT1*^+/y^ 1466 ± 171.4 μm^2^, *CT1*^-/y^ 524.6 ± 22.86 μm^2^, *n* = 3, **Figures 5C,D**). Given that *CT1*^-/y^ mice have severe thoracolumbar kyphosis and reduced muscle force during grip strength testing, both muscle dysfunction and atrophy are likely to be present in all major muscle groups.

### CT1 Deficiency Improves Glucose Tolerance

**FIGURE 6:**
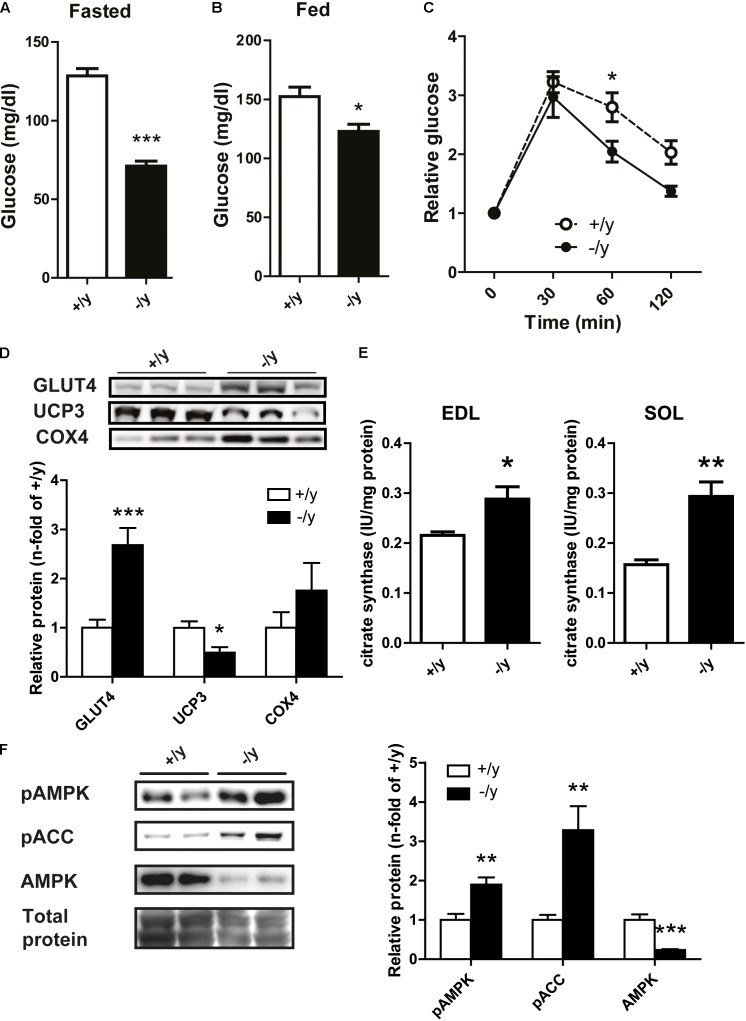
Glucose metabolism and protein expression. **(A,B)** Fasted and fed blood glucose levels in CT1^+/y^ and CT1^-/y^ mice. **(C)** Glucose tolerance test normalized to fasted blood glucose levels (*n* = 8–9). **(D)** Relative protein expression of muscle GLUT4, UCP3, and COX4 (*n* = 6). **(E)** Citrate synthase activity in *extensor digitorum longus* (left) and *soleus* (right) muscle (*n* = 4–5). **(F)** Protein levels of pAMPK, pACC, and AMPK in muscle tissue (*n* = 6–8) (^∗^*P* < 0.05; ^∗∗^*P* < 0.01; ^∗∗∗^*P* < 0.001).

In a previous study, we detected a pronounced metabolic phenotype in Cr-deficient AGAT^-/-^ mice that was characterized by improved glucose tolerance and resistance to diet-induced obesity (Choe et al., 2013b). Reduced body weight and altered muscle physiology suggested similar metabolic alterations in *CT1*^-/y^ mice. Indeed, fasted and fed blood glucose levels in *CT1*^-/y^ mice were significantly decreased (fasted: *CT1*^+/y^ 128.4 ± 4.67 mg/dl, *CT1*^-/y^ 71.13 ± 3.14 mg/dl, fed: *CT1*^+/y^ 152.3 ± 8.11 mg/dl, *CT1*^-/y^ 122.9 ± 6.08 mg/dl, **Figures 6A,B**). Furthermore, glucose tolerance tests revealed faster glucose clearance in *CT1*^-/y^ mice compared to *CT1*^+/y^ mice after normalization to fasted blood glucose levels (**Figure 6C**). As a large proportion of glucose is taken up into skeletal muscle, we analyzed the steady-state levels of glucose transporter protein type 4 in skeletal muscle. Quantitative western blot analysis revealed significantly increased GLUT4 levels in *CT1*^-/y^ mice (**Figure 6D**), which explains faster glucose clearance. In addition, we detected reduced expression levels of UCP3 and a trend toward increased COX4 expression indicating facilitated oxidative phosphorylation (**Figure 6D**). Furthermore, citrate synthase activity – a measure of mitochondrial mass – was significantly increased in *extensor digitorum longus* and *soleus* muscle (**Figure 6E**). These findings suggest mitochondrial content is increased to accelerate energy production to meet increased energy demand. Given the imbalance between energy demand and production, we looked for the activation of AMPK, a key metabolic energy sensor and switch. Similar to GAMT and AGAT mice, AMPK activation, as measured by phospho-AMPK (pAMPK) or phosphorylated substrate (pACC) levels, was increased in *CT1*^-/y^ mice indicating a chronic catabolic state, possibly resulting from energy deficiency, in skeletal muscle (**Figure 6F**).

### Reduced Phosphocreatine Levels and Altered Muscle Energy Metabolism in CT1-Deficient Mice

**FIGURE 7:**
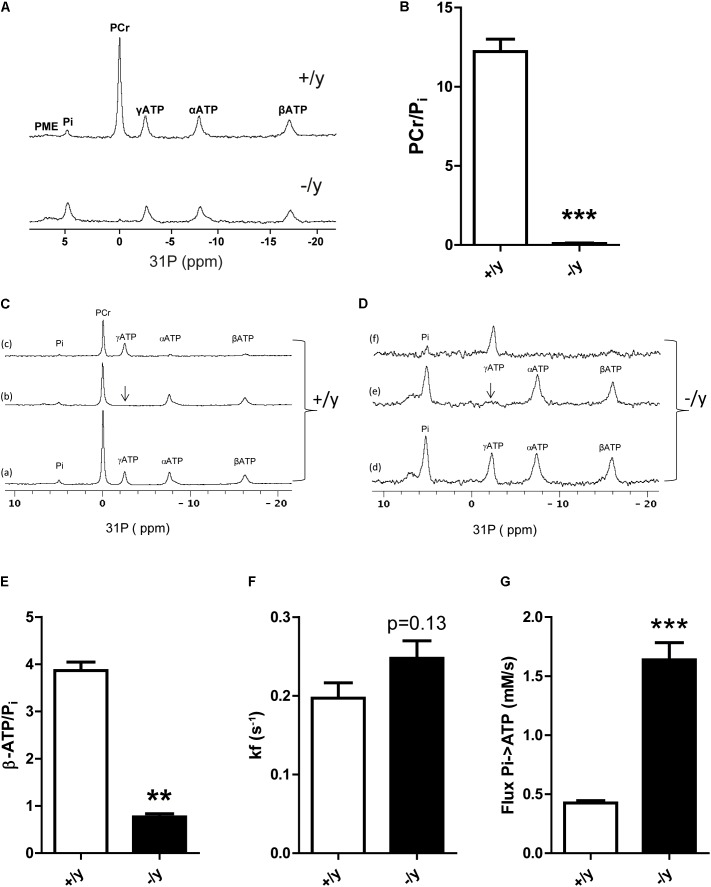
Muscle energy metabolism. **(A)** Representative ^31^P MR spectra of hind limb muscle in CT1^+/y^ and CT1^-/y^ mice (PCr, phosphocreatine; Pi, inorganic phosphate; PMEs, phosphomonoesters; ppm, parts per million). **(B)** PCr/P_i_ ratios in CT1^+/y^ and CT1^-/y^ mice measured with ^31^P MR spectroscopy (*n* = 4–5). **(C,D)** Saturation transfer ^31^P MRS experiments on hind limb muscle of CT1^+/y^ **(A)** and CT1^-/y^ mice **(B)** to assess the Pi→ATP kinetics. The γATP peak is saturated (b) and this saturation is transferred to the PCr and Pi peaks due to the creatine kinase and ATPase activity (subtraction spectra c = a minus b). This effect was used to calculate the forward rate constant *k*_f_. ATP to free phosphate ratios (ATP/Pi) **(E)**, *k*_f_ values **(F)**, and phosphate (Pi→ATP) flux **(G)** of hind limb muscle in CT1^+/y^ and CT1^-/y^ mice assessed with MR spectroscopy (*n* = 4–5) (^∗∗^*P* < 0.01; ^∗∗∗^*P* < 0.001).

Our data indicate profound metabolic changes in muscle of CT1-deficient mice. Therefore, we used muscle ^31^P MR spectroscopy to obtain functional data on high energy phosphate turnover. *CT1*^-/y^ mice demonstrated a strong reduction of PCr in skeletal muscle (**Figure 7A**). The PCr/P_i_ ratio was significantly reduced in *CT1*^-/y^ mice compared with CT1^+/y^ mice (CT1^+/y^: 12.2 ± 0.7, CT1^-/y^: 0.09 ± 0.02, *P* < 0.001, **Figure 7B**). Estimation of PCr tissue concentrations in skeletal muscle revealed a 40-fold reduction in *CT1*^-/y^ mice (*CT1*^+/y^: 25.4 ± 0.1 mM, *CT1*^-/y^: 0.6 ± 0.1 mM, *P* < 0.001). In contrast to this decrease, the Pi tissue concentration in skeletal muscle of CT1^-/y^ mice was increased threefold (*CT1*^+/y^: 2.16 ± 0.22 mM, *CT1*^-/y^: 6.6 ± 0.3 mM, *P* < 0.001). Furthermore, we determined whether ATP levels were decreased in the hind limb of CT1^-/y^ mice by evaluating the signal intensity of ATP relative to the muscle CSA (Nabuurs et al., 2013). Assuming a tissue concentration of 7.8 mM in wild-type skeletal muscle (in ’t Zandt et al., 2003), the ATP concentration in CT1^-/y^ hind limb muscle was estimated to be 4.2 ± 1.2 mM and therefore decreased by more than 40%. ATP/P_i_ ratios were significantly reduced in CT1^-/y^ mice, indicating abnormal energy metabolism by contributing to a decreased resting state phosphorylation potential due to the depletion of PCr stores (*CT1*^+/y^: 3.87 ± 0.16, *CT1*^-/y^: 0.77 ± 0.06, *P* < 0.001, **Figures 7C–E**). The calculated *in vivo* tissue pH was unaltered between *CT1*^+/y^ and *CT1*^-/y^ mice. Next, we assessed kinetics of ATP synthase in resting skeletal muscle from ST experiments. The forward rate *k*_f_ of ATP synthases in skeletal muscle of *CT1*^-/y^ mice was increased compared with *CT1*^+/y^ mice, but did not reach statistical significance (*CT1*^+/y^: 0.20 ± 0.04 s^-1^, *CT1*^-/y^: 0.25 ± 0.05 s^-1^, *P* = 0.13) (**Figure 7F**). The trend toward increased *k*_f_ of ATP synthase in *CT1*^-/y^ mice indicates increased total cytosolic and mitochondrial ATP synthetase activity. Given the increased Pi tissue concentration in *CT1*^-/y^ mice, the Pi→ATP flux is increased in *CT1*^-/y^ mice compared with *CT1*^+/y^ mice (*CT1*^+/y^: 0.43 ± 0.08 mM s^-1^, *CT1*^-/y^: 1.6 ± 0.3 mM s^-1^, *P* < 0.001) (**Figure 7G**). But, increased Pi→ATP flux as a parameter of total ATP synthase activity at rest is not sufficient to compensate for reduced ATP levels in the absence of PCr. Taken together, changes in muscle function and morphology of *CT1*^-/y^ mice were associated with marked alterations in energy metabolism, emphasizing the critical role of CT1 in muscle physiology.

## Discussion

Our study revealed that CT1-deficient mice have a strongly reduced skeletal muscle (P)Cr, an impaired motor function with severe muscle atrophy, and increased glucose metabolism due to AMPK activation. Major clinical problems in CT1-deficient patients include behavioral abnormalities, seizures, and intellectual impairment (Stockler et al., 2007). Therefore, previous research was mainly focused on the effects of Cr depletion in brain. In all available Cr-deficient mouse models, however, pronounced muscular phenotypes were observed (Schmidt et al., 2004; Nabuurs et al., 2013; Baroncelli et al., 2014; Russell et al., 2014). Here, we demonstrate strongly reduced PCr levels in skeletal muscle of CT1-deficient mice resulting in chronic energy depletion with increased resting Pi/ATP ratios. Muscle physiology, metabolism, and morphology were severely affected in CT1-deficient mice resulting in muscular atrophy and thoracolumbar scoliosis.

Following synthesis in liver and kidney, Cr delivery to its target organs such as muscle and brain is dependent on active transporters. To date, only CT1 encoded by *SLC6A8* has been identified as effective Cr transporter across cell membranes (Abplanalp et al., 2013; van de Kamp et al., 2014). Consequently, impaired CT1 activity has been associated with reduced cerebral and muscular Cr levels (deGrauw et al., 2003; van de Kamp et al., 2013). Here, we show that CT1 deficiency leads to markedly reduced Cr levels detected by HPLC-analysis and even stronger reduced PCr levels with *in vivo* ^31^P MR spectroscopy in skeletal muscle (**Figures 1, 7**). In previous studies on muscle of CT1-deficient mice, however, residual Cr was reported from below detection limit up to 23 or 36% of wild-type levels (Skelton et al., 2011; Russell et al., 2014; Baroncelli et al., 2016). In addition, two case studies on CT1-deficient patients showed almost normal levels of Cr in skeletal muscle (deGrauw et al., 2003; Pyne-Geithman et al., 2004). These discrepancies in muscular Cr content might be explained by residual CT1 activity in affected human patients (depending on the molecular nature of the mutation) or by mouse strain differences. The differences in Cr levels might also be due to specific detection techniques. Photometric assays can be unspecific and overestimate Cr values (Skelton et al., 2011; Russell et al., 2014).

An alternative explanation for residual Cr levels in muscle and brain of CT1-deficient animals as well as patients might be endogenous Cr synthesis. Under healthy or normal physiological conditions, AGAT levels are low in skeletal muscle (**Figure 2**). Cr synthesis is thought to take place almost exclusively in liver and kidney, with a minor contribution from pancreas (Wyss and Kaddurah-Daouk, 2000; Brosnan and Brosnan, 2010; da Silva et al., 2014). However, AGAT expression is regulated by Cr availability in a negative feedback loop (Choe et al., 2013a; Russell et al., 2014). Indeed, we detected increased AGAT expression in skeletal muscle following intracellular Cr depletion due to CT1 deficiency (**Figure 2**). As PCr levels remained low despite GAMT and increased AGAT expression, endogenous Cr synthesis in muscle was insufficient to compensate for CT1 deficiency in our mouse model. Thus, CT1 deficiency in mice induces a strong decrease in Cr in muscle, despite compensatory upregulation of enzymes involved in Cr synthesis.

Skeletal muscle contains Cr in concentrations of up to 40 mM. Accordingly, muscle physiology was severely affected in Cr-deficient CT1^-/y^ mice. Reduced muscle force and endurance as well as pronounced kyphoscoliosis demonstrate generalized impairment of muscle function (**Figure 5**). These functional alterations were accompanied by structural changes such as a reduction in muscle and myocyte diameter, which is in line with reports describing a close correlation of muscle diameter and contractile power (Maughan et al., 1983a,b). Absolute numbers of myocytes were not quantified, but similar reductions in muscle and myocyte diameters do not suggest a reduction in the overall number of myocytes. A reduced myocyte diameter can be explained by Cr deficiency itself and by chronic energy depletion and involvement of catabolic pathways including AMPK activation. Previous studies on AGAT- and GAMT-deficient mice showed ultrastructural changes in muscle histology, including lipid droplets around mitochondria (Schmidt et al., 2004; Nabuurs et al., 2013). Also AGAT-deficient muscle displayed a transcriptomic signature resembling a transcriptional response known as the mitochondrial unfolded protein response (Stockebrand et al., 2016; Shpilka and Haynes, 2018). Neither ultrastructural analysis of skeletal muscle nor transcriptomic analyses have been performed in CT1-deficient mice yet, but similar processes are likely to be involved.

Motor behavior in CT1-deficient mice was impaired not only in terms of muscle force but also wheel running distance and motor learning (**Figure 5**). Reduced wheel running might partly be explained by low muscle force, but also by deficient energy stores and impaired oxidative phosphorylation (see below). During rotarod testing for motor learning and coordination, performance during the first trial did not differ from CT1^+/y^ mice because low body weight might compensate for reduced muscle strength. However, unlike CT1^+/y^ mice, the performance of CT1^-/y^ mice did not improve during subsequent trials indicating not only peripheral muscular but also CNS contribution to the motor phenotype.

Severely altered structure and function of skeletal muscle were also observed in Cr-deficient AGAT knockout mice, whereas GAMT knockout mice displayed a milder muscular phenotype most likely due to high levels of guanidinoacetate that, although being a weak substrate for creatine kinase, can be phosphorylated and partly substitute for Cr (Schulze et al., 2003; Ensenauer et al., 2004; Kan et al., 2004). Some muscle properties derived from ^31^P MRS data show strikingly similar differences with WT between AGAT and CT1 knockouts: in both PCr was dramatically reduced with a concomitant decrease of ATP by 40%. This resulted in a metabolic remodeling including a fourfold increased Pi/ATP at an unchanged tissue pH (Nabuurs et al., 2013). The observed stronger reduction in PCr (40-fold) than in total Cr (10-fold) would be in agreement with a creatine kinase equilibrium shift due to the lower ATP level. Apparently, despite increased Pi→ATP flux the muscles of CT1^-/y^ mice cannot maintain normal ATP levels, even in the resting state. Reduced ATP and increased Pi levels result in chronic AMPK activation indicating a catabolic state favoring energy production. Accordingly, mitochondrial volume is increased and oxidative phosphorylation is accelerated, but not sufficient to normalize ATP levels. In line with these findings, creatine-deficient muscle in AGAT^-/-^ mice revealed increased absolute mitochondrial mass, but decreased respiratory chain enzyme activities per mitochondrial content (i.e., citrate synthase activity) (Nabuurs et al., 2013).

Both functional (reduction in grip strength: 70% for AGAT^-/-^ vs. 85% in CT1^-/y^) and structural changes (reduction in myocyte diameter: 35% for AGAT^-/-^ vs. 65% in CT1^-/y^) were even more pronounced in CT1^-/y^ mice compared with AGAT^-/-^ mice (**Figures 4A, 5D**) (Nabuurs et al., 2013). PCr levels in adult mice measured by MRS were low both in AGAT^-/-^ and CT1^-/y^ mice and cannot explain the differences. However, it has to be considered that mice from both lines were exposed to high maternal Cr levels during pregnancy and lactation. In AGAT^-/-^ mice, Cr can be taken up into the target organs by CT1 and may compensate for deficient endogenous Cr synthesis to a certain extent. In CT1 deficiency, a lack of functional Cr transporters prevents normalization of Cr stores in muscle, even following high serum Cr levels throughout intrauterine and postnatal development. Hence, intracellular Cr deficiency during early development seems to play a critical role for the outcome of genetic Cr deficiency.

Cerebral creatine deficiency in mice resulted in cognitive impairment and neuropathological abnormalities, which might also contribute to the muscle phenotype of systemic CT1-deficient mice (Kurosawa et al., 2012; Baroncelli et al., 2016). But skeletal muscle of mouse models deficient in muscle creatine kinase lacked burst activity, revealed altered intracellular calcium release and increased fatigue with tetanic stimulation (van Deursen et al., 1993; Steeghs et al., 1997, 1998; Dahlstedt et al., 2000). In the absence of PCr/Cr buffering, *in vivo* MRS studies of muscle energetics revealed that ATP levels, pH and Pi levels could not be maintained during ischemic conditions despite increased glycolytic ATP synthesis (in ’t Zandt et al., 1999, 2003; Nabuurs et al., 2013). Therefore, creatine deficiency in skeletal muscle *per se* impairs energy metabolism and motor function independent of neuronal dysfunction.

Limitations of our study are that we cannot rule out that developmental alterations are also responsible for impaired motor function. Given that mice with CT1 deficiency revealed age-related progressive cognitive impairment associated with brain aging, motor behavior could also be altered due to developmental disturbances (Baroncelli et al., 2016). Furthermore, in our systemic CT1-deficient mouse models, we are unable to clearly differentiate neuronal and muscular contributions to motor function. Given that brain-specific CT1 deficiency in mice recapitulates some metabolic and cognitive characteristics, a contribution of neuronal creatine deficiency to motor function is possible (Kurosawa et al., 2012; Baroncelli et al., 2016). But other mouse models with disrupted PCr/Cr buffering (i.e., deficiency of AGAT, cytosolic or mitochondrial creatine kinase) clearly link muscle creatine deficiency with motor dysfunction.

Although cognitive impairment and developmental delay are the hallmarks of human Cr deficiency syndromes, muscular hypotonia and myopathy were also described in patients with AGAT, GAMT, and CT1 deficiency (Stockler et al., 1996; Anselm et al., 2006; Nouioua et al., 2013). The overt muscular phenotype in Cr-deficient mouse models, however, has not been observed in humans. In contrast to mice, human patients still ingest Cr via their normal diet. However, oral Cr supplementation is only feasible in AGAT and GAMT deficiency, but proved to be ineffective in CT1 deficiency (Stockler et al., 2007). Expression of AGAT is upregulated in muscle of CT1-deficient mice (**Figure 2**). It is possible that this compensatory mechanism is sufficient to maintain endogenous Cr synthesis in human patients. To our knowledge, AGAT and GAMT expression in patients has not yet been directly assessed, for example by muscle biopsy. Although impaired skeletal muscle function does not seem to be a critical symptom in most CT1-deficient patients, low intracellular PCr/Cr energy stores might further compromise muscle function and increase the risk of muscle damage in situations of metabolic stress such as infections or intensified exercise in CT1-deficient patients. In summary, CT1 deficiency results in reduced levels of high energy phosphates (PCr, ATP), muscle weakness and atrophy, despite compensatory mechanisms of creatine and ATP biosynthesis.

## Author Contributions

MS and C-UC conceived the concept and designed the work. MS, AS, SH, DI, HL, CL, AN, and C-UC designed, conducted and analyzed metabolic phenotyping, and molecular biology experiments. DD and AH designed, conducted, and analyzed the MRS-experiments. MS and IH-B generated CT1 knockout mice. AS and C-UC designed, conducted, and analyzed behavioral experiments. MS and C-UC drafted the work. All authors critically revised the manuscript, approved the final version of the manuscript, and agree to be accountable for all aspects of the work. All persons designated as authors qualify for authorship, and all those who qualify for authorship are listed.

## Conflict of Interest Statement

The authors declare that the research was conducted in the absence of any commercial or financial relationships that could be construed as a potential conflict of interest.

## Abbreviations

AGAT: L-arginine:glycine amidinotransferase
AMPK: AMP-activated protein kinase
Cr: creatine
CSA: cross-sectional area
CT1: creatine transporter
GAMT: guanidinoacetate *N*-methyltransferase
GLUT4: glucose transporter 4
GLUT4: glucose transporter 4
*k*_f_: (pseudo-first order unidirectional) forward rate constant (for Pi→ATP)
MRS: magnetic resonance spectroscopy
PCr: phosphocreatine
(P)Cr: either phosphocreatine or creatine
Pi: inorganic phosphate
PMEs: phosphomonoesters
